# Personalized diets in prevention and treatment of metabolic associated diseases

**DOI:** 10.1186/1878-5085-5-S1-A138

**Published:** 2014-02-11

**Authors:** Viktor O Petrov, Taras V Chendey, Nadiya V Boyko

**Affiliations:** 1 R&D Centre of Molecular Microbiology and Mucosal Immunology, Uzhhorod National University, Uzhhorod, Ukraine; 2 Department of Hospital Therapy, Faculty of Medicine of Uzhhorod National University, Uzhhorod, Ukraine; 3 Zakarpattya Regional Clinical Cardiology Dispensary, Uzhhorod, Ukraine; 4 Cassovia Life Science, Kosice, Slovak Republic

## Background

Dietary intake alters specifically human gut microbiota and mucosal immune response. Inflammation is major initiating factor of metabolic associated diseases namely CVD, obesity and diabetes type II.

## The aim

was to provide the scientific background for the implementation of personalised diets for the prevention and treatment of metabolic associated diseases.

## Materials and methods

Classic and modern experimental systems, animal and cells models in preclinical investigation had been used. Methanolic extracts of edible plants: nettle, dill, kale, persimmon, pomegranate, and *Sideritis scardica* were administered to mice orally for two weeks (15 mg/200µl/day/mouse). Level of IgAs in gut contents and cytokine profiles in supernatants of fragment cultures of gut associated lymphoid tissues was measured by ELISA, and numbers of immune cells quantified by FACS. Human DCs derived from peripheral blood monocytes were exposed to plant extracts at 2 mg/ml for 6 and 24 hours. The expression of CD markers by DCs and levels of secreted cytokines were assessed before and after exposure to the extracts. Clinical trials had been performed in order to detect the early microbial and immunological biomarkers and efficacy of polyphenols reach extracts effect on CVD functions.

## Results

Edible plants induced a shift in expression of pro-/anti-inflammatory CD1a molecules on a surface of human DCs derived from peripheral blood monocytes. Commensal bacteria act on moDCs differentiation and activation by dose- and strain-specific manner, altering the expression of CD1a, NOD-2, RLH, PPARγ and RARα genes and cytokines production. Plant extracts and commensal microorganisms modulate the humoral and cellular mucosal immune response in GALT tissues on mice models differently regulating IgA secretion by peritoneal cavity B1/B2 cells and compartment of small intestine. Microbiological and immunological biomarkers of metabolic associated diseases as a result of evaluation of all the complexity diagnostic parameters had been found. Selected plants were able affect endothelial function, lipid profile and insulin sensitivity of patients with metabolic and cardiovascular diseases in randomised controlled trial.

## Conclusion

These results support the concept of potential implementation of personalised diets for the patients with metabolic associated diseases.

## Outlook and expert recommendations

The current challenge is to perform clinical intervention study according to EFSA demands and to criteria of evidence-based medicine to investigate efficacy of personalised diets developed from components and original recipes of traditional foods.

